# The association between the supply and utilization of community-based primary care and child health in a context of hospital-oriented healthcare system in urban districts of Guangdong, China: a panel dataset, 2014–2016

**DOI:** 10.1186/s12913-020-05193-7

**Published:** 2020-04-15

**Authors:** Zhuojun Luo, Yuanzhu Ma, Naiqi Ke, Shuyi Xu, Ruwei Hu, Nan Hu, Li Kuang

**Affiliations:** 1 Department of Health Administration, School of Public Health, Sun Yat-sen University, Guangzhou, 510080 China; 2 Guangdong Women and Children Hospital, Guangzhou, 511442 China; 3 School of Finance, Guangdong University of Finance and Economics, Guangzhou, 510320 China; 4 Department of Internal Medicine, Family and Preventive Medicine, and Population Health Sciences, University of Utah School of Medicine and Huntsman Cancer Institute, Salt Lake City, UT 84132 USA

**Keywords:** Utilization of primary care, Interprovincial migrants, Ecological study, Child health, Multilevel model

## Abstract

**Background:**

Since 2009, the Chinese government has been reforming the healthcare system and has committed to reinforcing increased use of primary care. To date, however, the Chinese healthcare system is still heavily reliant on hospital-based specialty care. Studies consistently show an association between primary care and improved health outcomes, and the same association is also found among the disadvantaged population. Due to the “hukou” system, interprovincial migrants in the urban districts are put in a weak position and become the disadvantaged. Therefore, the aim of this study is to investigate whether greater supply and utilization of primary care was associated with reduced child mortality among the entire population and the interprovincial migrants in urban districts of Guangdong province, China.

**Methods:**

An ecological study was conducted using a 3-year panel data with repeated measurements within urban districts in Guangdong province from 2014 to 2016, with 178 observations in total. Multilevel linear mixed effects models were applied to explore the associations.

**Results:**

Higher visit proportion to primary care was associated with reductions in the infant mortality rate and the under-five mortality rate in both the entire population and the interprovincial migrants (*p* < 0.05) in the full models. The association between visit proportion to primary care and reduced neonatal mortality rate was significant among the entire population (*p* < 0.05) while it was insignificant among the interprovincial migrants (*p* > 0.05).

**Conclusions:**

Our ecological study based in urban districts of Guangdong province found consistent associations between higher visit proportion to primary care and improvements in child health among the entire population and the interprovincial migrants, suggesting that China should continue to strengthen and develop the primary care system. The findings from China adds to the previously reported evidence on the association between primary care and improved health, especially that of the disadvantaged.

## Background

In the World Health Organization’s Declaration of Alma-Ata, primary care was identified as the key to health attainment by all [1]. The primary care system in China was established in the 1950s, and has been contributing to the reduction of communicable diseases burden and the improvement of public health [2]. Since the 1980s economic reform, a market-oriented healthcare system has gradually developed and hospital-based specialty care has prospered [3]. It was not until 2009 that the Chinese government launched a plan to reform the already distorted healthcare system and committed to reinforcing the primary care system [4]. Resources have been poured into primary care infrastructure building, primary care personnel training, improving health equity, and developing other supporting programs, including salary reforms, the Basic Public Health Service Package, universal health insurance coverage, and an essential drug program [5]. The government subsidies to primary care institutions increased from CNY 19.8 billion (USD 2.9 billion) in 2008 to CNY 157.7 billion (USD 23.7 billion) in 2016 [6]. However, people still rely on hospital-based specialty care with 41% of visits to hospitals in 2016 [7] even if primary care provides higher economic accessibility. The average cost of an outpatient visit to a primary care institution was about CNY 85.1 (USD 12.8), compared to CNY 245.5 (USD 36.9) per hospital outpatient appointment and CNY 8604.7 (USD 1293.3) per admission in 2016 [8]. As the first tier of the healthcare system in China, primary care institutions, provide preventive and basic health services. District hospitals provide specialized care and support the development of primary care institutions [9]. Both primary care physicians and specialists at hospitals can be accessed by walk-in patients. In some cities, the basic health insurance offers higher reimbursement rates for those who are referred to hospitals by primary care physicians. But still, primary care does not yet function fully as a gatekeeper to higher levels of health care in China. According to previous studies, building a comprehensive primary care system is the key to accessible, affordable and integrated care [2, 10–14].

With rapid urbanization, more people now live in urban districts. Compared with rural counties, urban districts enjoy more secondary and tertiary medical resources. Guangdong province, where our study was based, is one of the most developed regions in China. Urban districts in Guangdong province particularly enjoy rapid economic development. A large number of interprovincial migrants have flooded into urban districts of Guangdong province over the past decades in pursuit of job opportunities. According to the national census in 2010, the resident population of Guangdong province was 104.3 million with over 20% interprovincial migrants [15]. Since the Chinese national policy has long been set up on a household registration system (hukou), a locality-based scheme, migrants are often excluded from comprehensive health insurance, local benefits of education, public health services, and housing [16–20], which makes them vulnerable and susceptible to a special set of health risks [21]. Children and women are particularly affected by these social determinants of health. In this way, children and women from this disadvantaged context become more likely to suffer adverse health outcomes. Primary care is cheaper and provides greater geographical accessibility than hospitals. As a result, primary care can be more accessible especially for the disadvantaged such as the interprovincial migrants.

The Health and Family Planning Commission in Guangdong has also been enforcing policies for building a comprehensive healthcare system in which primary care institutions are the basis to provide maternal and child health services. In China, residents, including local residents and migrants, have been entitled to 14 basic public health services since 2016, including maternal and child health management in primary care institutions [22]. Besides, primary care institutions in Guangdong are also responsible for a range of services including premarital and preconception care, prenatal care, child care (ages 0–6), family planning, pregnancy health management, and follow-ups before delivery and after discharge. Most of the services are free of charge under the National Basic Public Health Service Package and can be accessed by everyone.

Studies have consistently shown an association between primary care (which is indicated by supply of primary care physicians, having a relationship with the source of primary care, or the receipt of important features of primary care) and improved health outcomes (measured by all-cause mortality, heart disease mortality, stroke mortality, infant mortality, low birth weight, life expectancy, and self-rated health at various levels of analysis) [10, 12]. The evidence is no longer confined mostly to developed countries, as studies show programs or tasks of primary care are associated with improved health in low- and middle-income countries [11, 23]. Studies also report that higher supply of primary care physicians is associated with significant improvements of various aspects of health in more socially disadvantaged areas and populations [10, 12]. Existing evidence includes reports from developed and undeveloped countries, but few studies from China. Addressing the association in China is essential to understanding whether efforts to strengthen the primary care system have the potential to produce measurable population health improvements not only among the general population but among the disadvantaged interprovincial migrants. It will provide evidence to continue strengthening the primary care system.

This study investigated whether greater supply and utilization of primary care was associated with reduced child mortality among the entire population and among the interprovincial migrants in urban districts of Guangdong province, China. The results will be particularly significant because hospital-based specialty care still dominates the healthcare system. The results may also provide evidence to support the expanding of the primary care system in China.

## Methods

### Study design

The study was an ecological analysis based on a 3-year (2014–2016) unbalanced panel dataset of urban districts in Guangdong province.

The analysis unit was the urban administrative district. There were 178 observations in three consecutive years (60 districts in 2014, 58 in 2015, and 60 in 2016 respectively, because of the changes in administrative divisions and urbanization). The Chinese government has a five-tier hierarchy (state, provincial, city, district/county and township level). Parallel to the administrative system is the healthcare system. There is no independent health administration at the township (town) level. So, district/county is the basic unit of health administration. District hospitals and primary care institutions are generally under the direction of the district health administration. District health administration provides essential support for developing the primary care system in the district.

### Variables

Community health centers (CHCs), township health centers (THCs), village clinics, and outpatient clinics are defined as “grass-root” healthcare institutions by the national health statistics information center, and these institutions are considered as the source of community-based primary care services [2, 9]. In our study, all grass-root healthcare institutions open for business in one urban district were defined as the source of community-based primary care services in that district and all district-level hospitals open for business in one urban district were defined as the source of hospital care in that district. The district healthcare institutions included both primary care institutions and district-level (secondary) hospitals.

The interprovincial migrants in this study were defined as those residing in Guangdong province without residence registration for the province.

#### Outcomes

Neonatal mortality rate (NMR), infant mortality rate (IMR), and under-five mortality rate (U5MR) among the entire population (the entire population including the interprovincial migrants) and among the interprovincial migrants were the primary outcomes of this study. These are essential indicators recognized by Millennium Development Goals and Sustainable Development Goal 3 targets, and are widely used in previous studies [11, 24–30].

#### Independent variables

The supply and utilization of primary care indicators included number of primary care physicians (PCPs) per 10,000 population and visit proportion to primary care institutions in the district.

Structural indicators of the healthcare system included number of physicians working in district-level hospitals per 10,000 population and a dummy variable reflecting whether there were tertiary hospitals in the district.

Economic and social confounding variables included GDP per capita (CNY 10,000/USD 1586.34), population density (1000 people per square kilometer), percentage of children aged between 0 and 6, and percentage of pregnant women with rural registration. These confounding variables described the characteristics of a typical urban district in Guangdong province.

The definitions and sources of each variable are shown in Table 1.

**Table 1:** The definitions and data sources of the independent variables and outcomes

	Variable	Definition	Data source
The supply and utilization of primary care	Number of PCPs per 10,000 population	The number of physicians working in primary care institutions per 10,000 population. It indicated the supply of primary care in the district.	Health Statistical Bureau of Guangdong Province
	Visit proportion to primary care, %	The number of visits to primary care institutions as a percentage of visits to all district healthcare institutions. It showed the utilization of primary care services by people in the district.	
Structural indicators of healthcare system	Number of physicians working in district-level hospitals per 10,000 population	The number of physicians working in district-level (secondary) hospitals per 10,000 population. It indicated the supply of care in district-level hospitals.	
	Whether there were tertiary hospitals in the district	A dummy variable, coded with ‘1’ indicating one or more tertiary hospitals and ‘0’ indicating no tertiary hospital in the district.	
Confounders	GDP per capita (10,000 Yuan/USD 1586.34)	Division of gross domestic product (GDP) by the number of population in the district. It was a proxy for economic development in the district.	Statistical Yearbooks of 12 prefectural-level cities of Guangdong province
	Population density (1000 per square kilometer)	Population per square kilometer living in the district. It was a proxy for environment in the district.	
	Children aged between 0 to 6, %	The number of children aged between 0 and 6 as a percentage of the whole population in the district. It was a proxy for demographics in the district.	Annual national report of maternal and child health
	Pregnant women with rural registration, %	Pregnant women with rural registration as a percentage of all the pregnant women in the district. It was a proxy for demographics in the district.	
Child health indicators	U5MR (among the entire population/the interprovincial migrants)	The number of deaths of children under the age of five per 1000 live births among the entire population/the interprovincial migrants in the district.	Annual national report of maternal and child health
	IMR (among the entire population/the interprovincial migrants)	The number of deaths of infants under the age of one per 1000 live births among the entire population/the interprovincial migrants in the district.	
	NMR (among the entire population/the interprovincial migrants)	The number of deaths of infants during the first 28 days of life per 1000 lives births among the entire population/the interprovincial migrants in the district.	

### Assessment framework

**Fig. 1:**
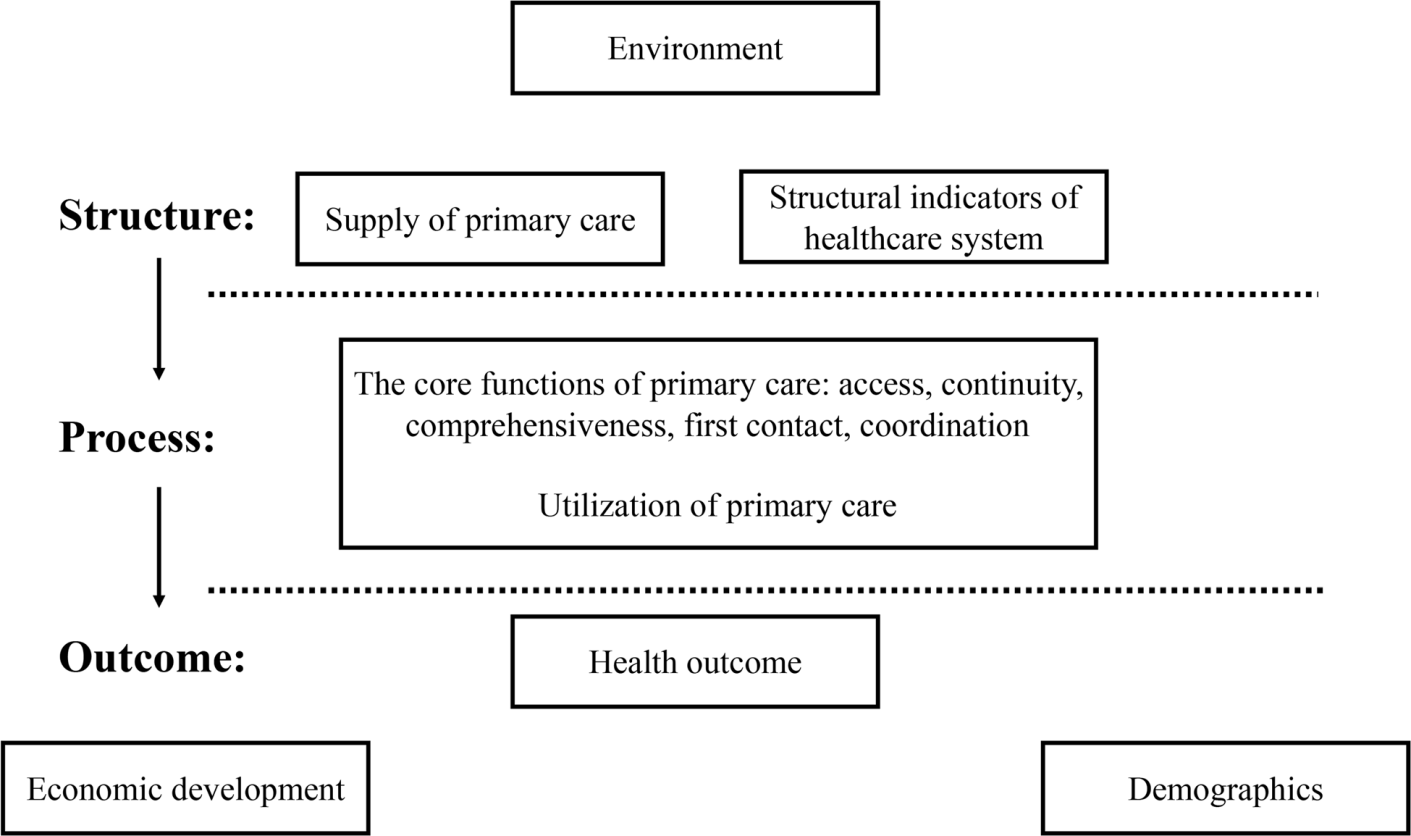
The assessment framework

Based on Donabedian model [31] and the studies of Starfield, [32] Macinko, [23] and Kringos, [33] we developed the assessment framework shown in Fig. 1.

Health care was produced by system inputs, the structure part (the supply of physicians and other structural indicators of healthcare system) that interacted with the population through process (the utilization of service) and resulted in health outcomes [34]. We focused on the association between the supply and utilization of primary care and health outcomes because of accumulated evidence of greater primary care associated with improved population health. GDP per capita and population density were proxies for economic development and environment, respectively. Percentage of children aged between 0 and 6 and percentage of pregnant women with rural registration were proxies for demographics.

### Statistical analysis

We performed the longitudinal analysis using panel data with repeated measurements within each urban district. Considering the nested nature of repeated measurements, multilevel linear mixed effects model was used, with a random intercept at the district level. Independent variables, including the supply and utilization of primary care indicators, structural indicators of the healthcare system, and other potential confounders were treated as fixed effects. Year was also treated as a fixed effect in the model. The primary outcomes (NMR, IMR, and U5MR) were expressed as rates, which were used in previous studies [28, 35]. All analyses were performed using statistical package R (http://www.r-project.org), version 3.4.4 [36].

Independent variables were added into the model step by step. In model 1, only the supply and utilization of primary care indicators were included as the exposures. In model 2, structural indicators of the healthcare system were added as the potential confounders. In model 3, extra confounding variables (economic and social confounders) were added.

All tests are two-sided. *P* value less than 0.05 indicates statistically significant results.

## Results

**Fig. 2:**
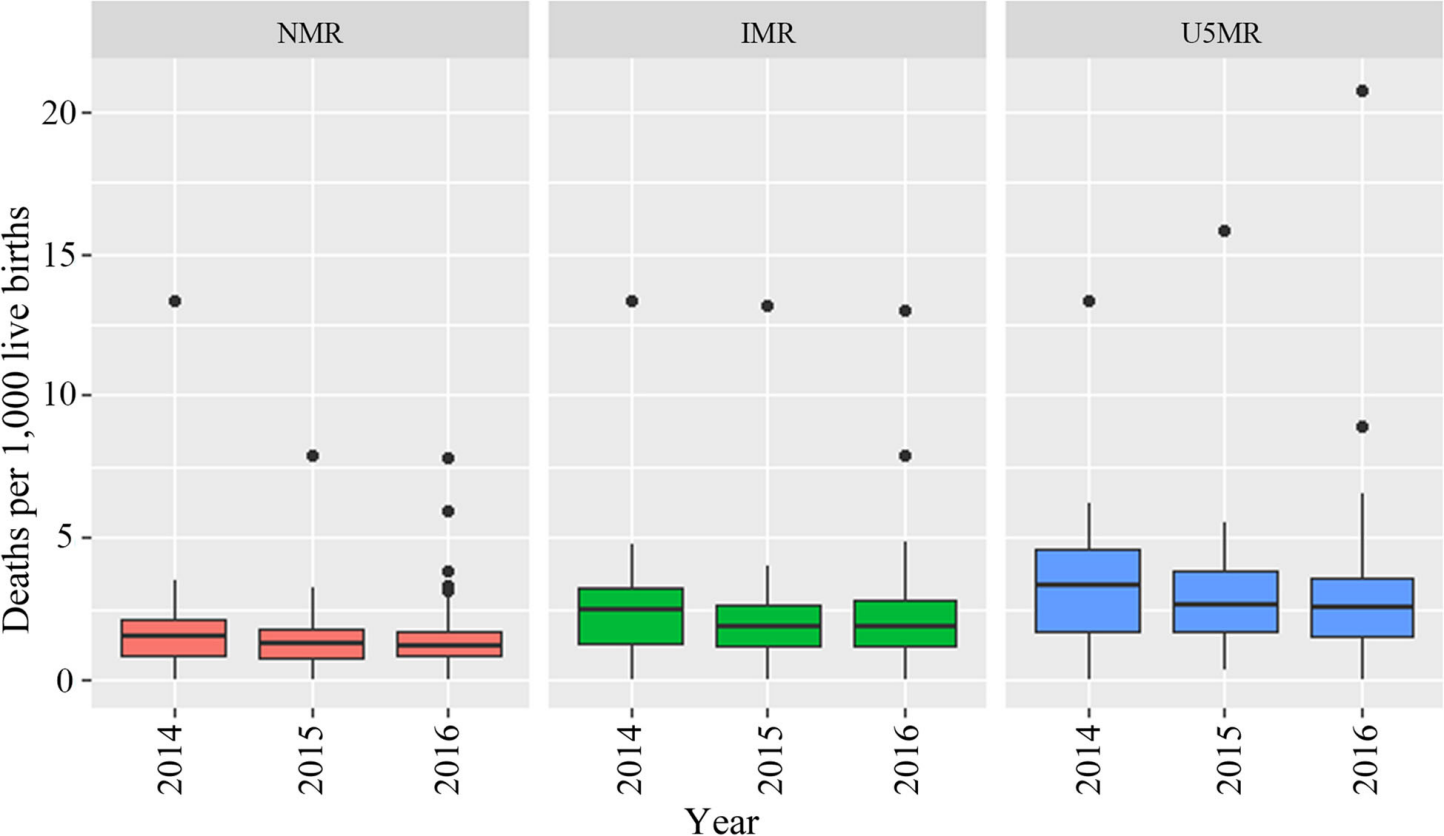
NMR, IMR, and U5MR among the entire population in urban districts of Guangdong province

**Fig. 3:**
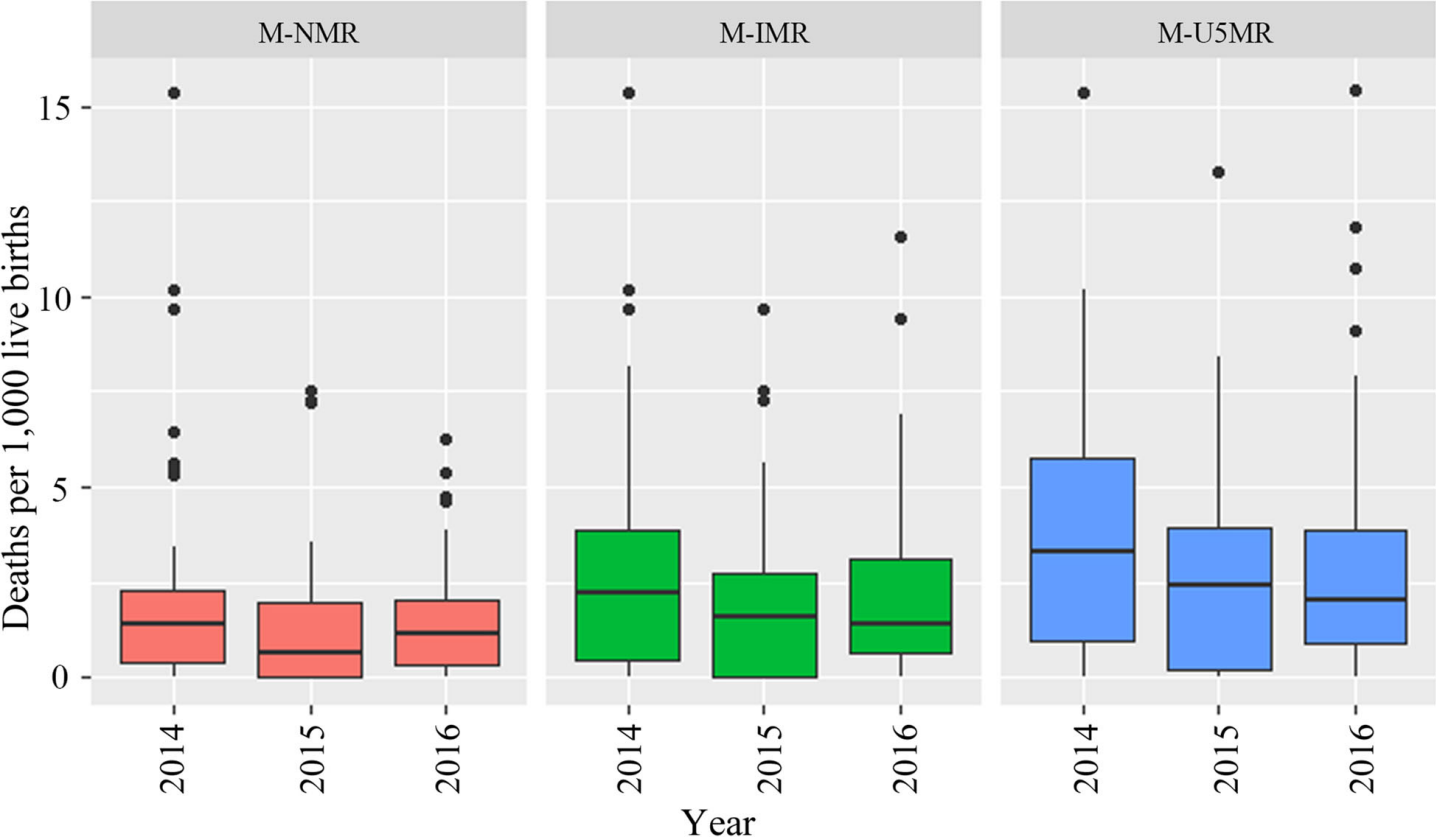
NMR, IMR, and U5MR among the interprovincial migrants in urban districts of Guangdong province (M-NMR, M-IMR, and M-U5MR stand for NMR, IMR, and U5MR among the interprovincial migrants respectively)

Descriptive statistics are shown in Table 2. From 2014 to 2016 in urban districts of Guangdong province, there was an increase for both the number of PCPs per 10,000 population and the number of physicians working in district-level hospitals per 10,000 population, from about 8.73 to 9.67 and 4.62 to 4.96 per 10,000 population, respectively. Visit proportion to primary care was stable at around 71%. Among the entire population in the district, the median of NMR, IMR, and U5MR first decreased and then remained relatively steady in the following year. The distributions were similar among the boxplots of NMR during the years. Seventy-five percent of the districts had IMRs below 3.75 (per 1000 live births), and U5MRs below 5 (per 1000 live births) during the period. Among the interprovincial migrants, NMR fluctuated during the years, and both IMR and U5MR declined from 2014 to 2016. The variability in U5MR of 3 years, as measured by the interquartile range, was relatively larger (Figs. 2 and 3).

**Table 2:** Descriptive statistics of the independent variables and outcomes

	2014 (*n* = 60)		2015 (*n* = 58)		2016 (*n* = 60)		Across all years (*n* = 178)			
	Mean	Std. Deviation	Mean	Std. Deviation	Mean	Std. Deviation	Mean	Std. Deviation	Min.	Max.
Number of PCPs per 10,000 population	8.73	3.68	9.11	3.44	9.67	3.81	9.17	3.65	2.38	19.30
Visit proportion to primary care, %	71.03	19.84	71.17	19.59	70.25	19.68	70.81	19.60	25.43	100.00
Number of physicians working in district-level hospitals per 10,000 population	4.62	3.44	4.71	3.55	4.96	3.65	4.77	3.53	0.00	15.38
GDP per capita (CNY 10,000/USD 1586.34)	8.11	7.09	9.29	6.80	9.51	6.90	8.96	6.92	1.50	46.56
Population density (1000 per square kilometer)	4.52	7.40	4.40	6.53	4.61	6.75	4.51	6.87	0.13	35.93
Children aged between 0 and 6, %	7.00	2.00	6.58	2.71	6.90	3.42	6.83	2.76	1.41	24.06
Pregnant women with rural registration, %	49.93	30.98	47.40	29.52	49.53	28.31	48.97	29.48	0.00	100.00
Resident population of the district (in thousands)	1262.45	1262.22	1356.53	1774.72	1303.49	1551.67	1306.94	1643.12	170.50	9843.06

Tables 3 and 4 show the results of multilevel linear models for NMR, IMR, and U5MR among the entire population and among the interprovincial migrants. As variables were added into the model, visit proportion to primary care became statistically significant and was significant for NMR, IMR, and U5MR among the entire population (*p* < 0.05), and for IMR and U5MR among the interprovincial migrants (*p* < 0.05) in model 3. In addition, the magnitude of the visit proportion to primary care coefficient increased each time other variables were added.

**Table 3:** Multilevel model results of NMR, IMR, and U5MR among the entire population in urban districts of Guangdong province

Variable	NMR			IMR			U5MR		
	Model 1	Model 2	Model 3	Model 1	Model 2	Model 3	Model 1	Model 2	Model 3
Number of PCPs per 10,000 population	0.003 (0.039)	0.047 (0.043)	0.024 (0.044)	−0.015 (0.048)	0.041 (0.055)	0.006 (0.055)	−0.006 (0.065)	0.088 (0.073)	0.052 (0.074)
Visit proportion to primary care, %	−0.009 (0.009)	− 0.033^b^ (0.014)	− 0.038^b^ (0.015)	− 0.008 (0.011)	− 0.038^b^ (0.018)	− 0.041^b^ (0.018)	−0.011 (0.015)	− 0.061^b^ (0.024)	−0.068^a^ (0.025)
Number of physicians working in district-level hospitals per 10,000 population	–	−0.148^b^ (0.070)	−0.179^b^ (0.072)	–	− 0.182^b^ (0.087)	−0.223^b^ (0.087)	–	− 0.306^a^ (0.117)	−0.366^a^ (0.118)
Tertiary hospitals in the district	–	−0.046 (0.217)	0.082 (0.221)	–	−0.196 (0.259)	−0.095 (0.255)	–	− 0.286 (0.349)	−0.151 (0.351)
GDP per capita (CNY 10,000/USD 1586.34)	–	–	0.011 (0.020)	–	–	0.026 (0.025)	–	–	0.021 (0.034)
Population density (1000 per square kilometer)	–	–	−0.028 (0.023)	–	–	−0.030 (0.029)	–	–	− 0.047 (0.039)
Children aged between 0 and 6, %	–	–	0.049 (0.030)	–	–	0.106^a^ (0.034)	–	–	0.116^b^ (0.047)
Pregnant women with rural registration, %	–	–	0.009 (0.006)	–	–	0.010 (0.007)	–	–	0.010 (0.010)
2015	−0.304^b^ (0.137)	−0.296^b^ (0.137)	− 0.261 (0.140)	−0.407^a^ (0.155)	− 0.396^b^ (0.154)	−0.352^b^ (0.154)	− 0.478^b^ (0.214)	−0.460^b^ (0.211)	− 0.406 (0.214)
2016	−0.186 (0.143)	− 0.174 (0.150)	−0.179 (0.150)	− 0.200 (0.163)	−0.159 (0.171)	− 0.167 (0.167)	−0.290 (0.225)	− 0.230 (0.232)	−0.234 (0.232)
Constant	2.306^a^ (0.588)	4.361^a^ (1.146)	4.205^a^ (1.293)	3.190^a^ (0.781)	5.744^a^ (1.428)	5.121^a^ (1.569)	4.116^a^ (1.021)	8.369^a^ (1.906)	8.158^a^ (2.125)
Observations	178	178	178	178	178	178	178	178	178

^a^ 1% ^b^ 5% Standard errors are in the parentheses

**Table 4:** Multilevel model results of NMR, IMR, and U5MR among the interprovincial migrants in urban districts of Guangdong province

Variable	M-NMR			M-IMR			M-U5MR		
	Model 1	Model 2	Model 3	Model 1	Model 2	Model 3	Model 1	Model 2	Model 3
Number of PCPs per 10,000 population	−0.042 (0.053)	−0.011 (0.058)	−0.008 (0.059)	− 0.075 (0.066)	−0.019 (0.074)	− 0.007 (0.076)	−0.113 (0.082)	− 0.038 (0.091)	−0.009 (0.093)
Visit proportion to primary care, %	−0.005 (0.010)	−0.018 (0.020)	− 0.032 (0.021)	−0.010 (0.013)	− 0.040 (0.025)	−0.053^b^ (0.026)	− 0.015 (0.016)	−0.054 (0.031)	− 0.070^b^ (0.032)
Number of physicians working in district-level hospitals per 10,000 population	–	−0.082 (0.102)	− 0.139 (0.104)	–	−0.185 (0.127)	− 0.234 (0.132)	–	−0.237 (0.156)	− 0.312 (0.162)
Tertiary hospitals in the district	–	0.539 (0.360)	0.831^b^ (0.378)	–	0.484 (0.435)	0.751 (0.457)	–	0.824 (0.532)	1.048 (0.557)
GDP per capita (CNY 10,000/USD 1586.34))	–	–	−0.032 (0.031)	–	–	−0.028 (0.038)	–	–	− 0.019 (0.046)
Population density (1000 per square kilometer)	–	–	− 0.046 (0.030)	–	–	−0.063 (0.039)	–	–	− 0.107^b^ (0.048)
Children aged between 0 and 6, %	–	–	−0.012 (0.062)	–	–	− 0.073 (0.071)	–	–	−0.110 (0.086)
Pregnant women with rural registration, %	–	–	0.006 (0.008)	–	–	0.006 (0.010)	–	–	0.002 (0.012)
2015	−0.758^b^ (0.345)	−0.776^b^ (0.344)	− 0.733^b^ (0.348)	−0.955^a^ (0.368)	− 0.965^a^ (0.366)	−0.952^b^ (0.369)	− 0.858 (0.444)	−0.878^b^ (0.439)	− 0.914^b^ (0.441)
2016	−0.555 (0.346)	− 0.674 (0.353)	−0.674 (0.356)	− 0.595 (0.372)	−0.701 (0.381)	− 0.713 (0.382)	−0.556 (0.449)	− 0.735 (0.458)	−0.767^b^ (0.458)
Constant	2.770^a^ (0.721)	3.566^b^ (1.707)	4.928^b^ (1.933)	4.148^a^ (0.915)	6.444^a^ (2.098)	8.035^a^ (2.413′)	5.624^a^ (1.135)	8.431^a^ (2.577)	10.932^a^ (2.955)
Observations	178	178	178	178	178	178	178	178	178

^a^ 1% ^b^ 5% Standard errors are in the parentheses. M-NMR, M-IMR and M-U5MR stand for NMR, IMR and U5MR among the interprovincial migrants respectively

For the entire population, with every one unit increase in visit proportion to primary care, NMR, IMR, and U5MR decreases 0.038 (per 1000 live births), 0.041 (per 1000 live births) and 0.068 (per 1000 live births) respectively while other variables were held constant in the full model. The number of physicians working in district-level hospitals per 10,000 population was significant in model 3 for NMR, IMR, and U5MR (*p* < 0.05). With every one unit increase in the number of physicians working in district-level hospitals per 10,000 population, NMR, IMR, and U5MR decreases 0.179 (per 1000 live births), 0.223 (per 1000 live births) and 0.366 (per 1000 live births) respectively while other variables were held constant in the full model.

For the interprovincial migrants, an increase of one unit of the visit proportion to primary care was associated with 0.053 (per 1000 live births) reduction in IMR and with 0.070 (per 1000 live births) reduction in U5MR while other variables were held constant. For NMR, neither of the primary care indicators were significant. The number of physicians working in district-level hospitals per 10,000 population did not achieve significance level for any child health outcomes among the interprovincial migrants.

All independent variables used in the models were checked for multicollinearity, which was not found (results not shown).

## Discussion

Few studies have investigated the association between greater supply and utilization of primary care and child health in urban areas of China. By conducting an ecological study using a district-level panel dataset of Guangdong province, our study intended to fill the gap and add to the existing evidence regarding the association. Significant associations were found between visit proportion to primary care and child health among the entire population and the interprovincial migrants in urban districts of Guangdong province. The strength of the association increased when structural indicators of the healthcare system and confounders were added into the models. This may reflect the degree of association between visit proportion to primary care and child health remained after the effects of the structural indicators and economic and social confounders have been controlled. It provided a more appropriate estimate of the true association.

Visit proportion to primary care was statistically and negatively associated with child health outcomes among the entire population in the fully adjusted model, adding to the existing evidence supporting the positive effects of primary care on health. Past studies have shown similar results. Features of primary care, such as continuity [24], comprehensiveness [24, 25, 28–30], accessibility [11, 25, 28–30], coordination of care [30], and community-based services [30] may underlie the improved population health evident in our results.

The number of physicians working in district-level hospitals per 10,000 population was negatively associated with NMR, IMR, and U5MR among the entire population. Goodman and Grumbach concluded that the supply of physicians other than PCPs was weakly associated with health outcomes [37]. Other studies have suggested that specialty care was either not associated with mortality or was associated with poorer health outcomes in the US [38, 39]. However, the relationship of reduced child mortality in our study was consistent with studies finding that the technical skills of PCPs still lag behind that of hospital physicians during the transformation of the hospital-oriented healthcare system in China. Also, hospitals have better equipment and better capability to handle emergencies [2, 40–42]. Thus, hospital-based specialty care may be associated with reduced mortality. That being said, hospitals and specialty care alone are not a guarantee of success and may hinder efforts to establish an accessible, affordable, patient-centered and integrated healthcare system [12, 37, 43, 44].

Our study found a negative association between visit proportion to primary care and child mortality among the interprovincial migrants. Past research has reported primary care was associated with improved health of the disadvantaged (i.e., minorities and the poor) [11, 12, 27, 38, 45–47]. The finding that the number of physicians working in district-level hospitals per 10,000 population was not significantly associated with reduced child mortality among the interprovincial migrants further suggests that primary care rather than hospital-based specialty care was associated with improved health of the disadvantaged. This may be explained by the financial and geographical accessibility, and the comprehensiveness of primary care [11, 27, 38, 48]. However, there are studies indicating that interprovincial migrants’ utilization of healthcare service is lower compared to that of local district residents in the district [17, 18, 21, 49, 50]. Since the two primary care indicators did not fully capture the interprovincial migrants’ true utilization rate of services, combined with their reported underuse of healthcare service, our results may underestimate the association between greater utilization of primary care and improved health of the interprovincial migrants.

Notably, we did not find a significant association between NMR and primary care among the interprovincial migrants. Although neonatal mortality is expected to be influenced indirectly by primary care through the promotion of maternal health and nutrition, prenatal care, and referral of potentially high-risk births to hospitals, it relates more to conditions and diseases associated with lack of quality care during delivery and post-delivery in hospitals [51]. A study carried out in Jiangsu province found that migrants were about 5 times less likely to attend prenatal examinations, 3 times less likely to pay postnatal visits and also less likely to accept health education during pregnancy compared with local residents [17]. This may also be a possible explanation.

GDP per capita in our study was not a significant predictor for health outcomes, despite that it has been widely used in similar studies as an indicator of economic growth. In China, the government has always attached great importance to child health. Guangdong province published the Outline for the Development of Children in China in 2011, setting targets to control child mortality [52]. Throughout the province, every district government has committed to establishing a fair system of health care for women and children. Therefore, it might be reasonable that GDP per capita had no association with child health because improving child health has been a mandatory target regardless of a district’s economic development.

Descriptive statistics showed that the growth rate of PCPs per 10,000 population (from 8.73 to 9.67) was larger than the growth rate for district-level hospital physicians (4.62 to 4.96). There was no corresponding growth rate for visit proportion to primary care. National statistics show a similar trend across the country [7, 53], indicating an underuse of primary care services although more human resources were allocated. This is one of the challenges facing primary care in China. Under a hospital-oriented system without gate-keeping and effective referral mechanism, patients are free to seek care in hospitals. Based on accumulated evidence for the positive association between greater primary care and improved health, we should recognize the positive effects of primary care and increase measures that encourage patients to seek primary care rather than hospital-based care.

### Limitations

First, this was an ecological study, so we made inferences on the district level; the results cannot be generalized to the individual level or other levels. At the same time, it was a correlational study and correlation did not necessarily imply causation. All the results should be inferred with caution. Given the unavailability of data, we cannot obtain the actual number of PCPs per 10,000 interprovincial migrants or the visit proportion to primary care among them. We did not account for the proportion of interprovincial migrants in the entire population. However, we used indicators of the entire population as proxies for the interprovincial migrants, since primary care institutions are rooted in the community context and migrants have equal access as local residents.

Next, the two indicators of primary care only reflected the degree of primary care resource supply and service utilization, and did not reflect whether people actually received qualified primary care services. At the same time, we did not use indicators related to features of primary care (first-contact, access, continuity, comprehensiveness, coordination, etc.), which may reflect the core functions of primary care and explain the contributory mechanism of primary care [10, 23]. Therefore, the magnitude of association in our results may be an underestimate. Besides, our study samples were all urban districts of Guangdong province, so the results are valid only for Guangdong province. It did not necessarily generalize to other provinces of China.

Finally, in this study we only focused on child health outcomes. The potential association between primary care, or core functions of primary care and chronic diseases, life quality, and disease burden remains largely unexplored. Further studies may use more comprehensive health indicators to explore the relations between primary care and population health. As the Chinese healthcare system is further reformed, more data are expected to be available and longer panels can be made. Until then, a more significant positive effects of primary care on population health may be found and causal assumptions might be tested.

## Conclusions

Guangdong province has been strengthening its primary care system, and the improvements are tangible. Our study showed a positive association between visit proportion to primary care and child health within an urban district-level context where the healthcare system is dominated by hospital-based specialty care. The association was significant among the interprovincial migrants as well, providing additional evidence for greater utilization of primary care associated with improved health. With policies that encourage primary care and with increased availability of community-based primary care systems in urban districts, primary care will exhibit greater association with population health, inviting further exploration. We should continue to encourage the use of primary care and strive to make it the central part of the healthcare system in China.

## Data Availability

The data that support the findings of this study are available from the Health Statistics Bureau of Guangdong province but restrictions apply to the availability of these data, which were used under license for the current study, and so are not publicly available.

## Abbreviations

CHC: Community health center
IMR: Infant mortality rate
M-IMR: Infant mortality rate among the interprovincial migrants
M-NMR: Neonatal mortality rate among the interprovincial migrants
M-U5MR: Under-five mortality rate among the interprovincial population
NMR: Neonatal mortality rate
PCP: Primary care physician
THC: Township health center
U5MR: Under-five mortality rate
